# *In‐vitro* Assessment of BCRP‐Mediated Efflux of Antiseizure Medications in Human Blood‐Brain Barrier Cell Model

**DOI:** 10.1002/jbt.70570

**Published:** 2025-10-27

**Authors:** Shivangi Bora, Priyanka Rani Paul, Samiksha Kukal, Manish Kumar Mishra, Yasha Hasija, Ritushree Kukreti

**Affiliations:** ^1^ Department of Biotechnology Delhi Technological University Shahbad Daulatpur Delhi India; ^2^ Genomics and Molecular Medicine Unit Institute of Genomics and Integrative Biology (IGIB), Council of Scientific and Industrial Research (CSIR) Delhi India; ^3^ Academy of Scientific and Innovative Research (AcSIR) Ghaziabad India

**Keywords:** antiseizure medications, BCRP, drug‐resistant epilepsy, hCMEC/D3

## Abstract

Drug‐resistant epilepsy (DRE), where current antiseizure medications (ASMs) are ineffective in controlling seizures, affects approximately one‐third of epilepsy patients. One potential mechanism that explains DRE is the presence of efflux transporters, like breast cancer resistance protein (BCRP) at the blood‐brain barrier (BBB), that hamper the exposure of several ASMs to the brain. Here, we employed ATPase assay, competitive substrate efflux assay, and bidirectional transport assay to explore the interaction of BCRP with frequently prescribed eight ASMs. Immortalized human cerebral microvascular endothelial cells (hCMEC/D3) were used as a human BBB cell model. The obtained ATPase assay data revealed N‐desmethyl clobazam at 12 µM and oxcarbazepine at 40 µM stimulated baseline ATPase activity of BCRP (*p* < 0.05). They also influenced the BCRP‐mediated BODIPY‐prazosin efflux in competitive substrate efflux assay, since N‐desmethyl clobazam (1 µM & 10 µM) and oxcarbazepine (12 µM & 140 µM) increased the BODIPY‐prazosin intracellular accumulation (*p* < 0.01). Bidirectional transport experiments demonstrated significant directional transport of N‐desmethyl clobazam (efflux ratios: 2.0 at 5 µM & 10 µM), and oxcarbazepine (efflux ratios: 1.81 & 2.37 at 25 µM & 140 µM). Co‐incubation with the BCRP inhibitor significantly reduced the efflux ratios for these two ASMs (*p* < 0.01), confirming active BCRP‐mediated efflux of N‐desmethyl clobazam and oxcarbazepine. Collectively, these findings provide evidence that among eight ASMs, N‐desmethyl clobazam, and oxcarbazepine may be transported by BCRP at clinically relevant concentrations, and targeting BCRP may potentially enhance future epilepsy treatments.

## Graphical abstract

Efflux transporter ABCG2/BCRP is reported to be overexpressed in drug‐resistant epilepsy that results in decreased bioavailability of antiseizure medications (ASMs) that are its substrates. Interaction of ASMs with BCRP was investigated using ATPase, substrate efflux assay and bidirectional transport assay. N‐desmethyl clobazam and oxcarbazepine have been identified as BCRP substrates.

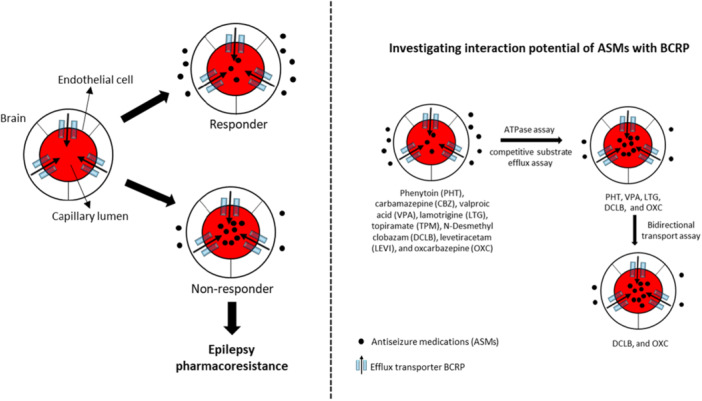

## 1 Introduction

Epilepsy, a common neurological disease, affects approximately 50 million people globally and faces a significant challenge of pharmacoresistance [1, 2]. Although around 35 antiseizure medications (ASMs) are currently in clinical practice [3], both conventional and new‐generation ASM therapy encounter this challenge, which accounts for 30% of total epilepsy cases [4, 5, 6, 7]. This condition is referred to as drug‐resistant epilepsy (DRE) and is defined as the failure of appropriately chosen two ASMs either as monotherapy or in combination, to attain control over seizures [8]. However, mechanisms underlying DRE are not understood clearly and are likely to have both genetic and environmental influences [9, 10].

Most ASMs penetrate through the BBB into the brain via passive diffusion [11], but the presence of ATP‐binding cassette (ABC) efflux transporters like breast cancer resistance protein (BCRP), P‐glycoprotein (P‐gp), and multidrug resistance‐associated proteins may cause ASM expulsion limiting its bioavailability [12, 13, 14, 15, 16, 17]. The primary physiological function of ABC efflux transporters is to protect organs from toxins and xenobiotics [18]; however, in‐vivo and clinical studies have shown that overexpression of these transporters in brain tissues of animal models and patients of DRE hinders ASM efficacy by transporting them back into blood circulation [15, 17, 19, 20, 21]. Previous reports utilizing rat models of DRE showed certain ASMs are effluxed by ABC efflux transporter like P‐gp and its inhibition reverses the resistance to ASM that are its substrates [22, 23]. This highlights the prominent role of the transporter hypothesis in DRE.

P‐gp has been extensively explored, however, studies on BCRP‐mediated ASM efflux or brain distribution are limited and often yield contradictory results. The study by Cerveny et al. concluded that human BCRP does not transport any ASM using a human BCRP‐transfected MDCKII cell line [24]. However, another in‐vitro report showed an ASM to be BCRP substrate utilizing the same cell line [25]. Also, research involving genetically modified mice lacking either P‐gp or Bcrp [21], indicated that both P‐gp and Bcrp restrict the brain access of several ASMs. The discrepancy between findings of in‐vitro reports might be attributed to the use of a canine kidney tissue‐derived cell line instead of a more specific human brain‐derived cell line. This emphasizes the necessity for a standardized and thorough method to investigate ASMs that interact with BCRP through multiple in‐vitro assays, utilizing human‐derived cells [26, 27, 28, 29]. This may lead to a better understanding of the role of BCRP in the DRE mechanism. A recent in‐vivo study suggests that BCRP‐mediated efflux impacts ASM efficacy, leading to poor seizure management [30]. ASMs classified as BCRP substrates undergo increased efflux at the BBB, reducing their CNS concentrations and ultimately diminishing seizure control. This underscores the necessity of assessing BCRP substrate status when selecting ASMs to optimize seizure management.

In the literature, techniques such as ATPase assay and competitive substrate efflux assay, are valuable in determining interaction between ASMs and BCRP [31]. In addition, the bidirectional transport assay is regarded as the optimal method of choice to demonstrate if a compound is actively effluxed out by BCRP [31, 32]. Along with these techniques, choosing an appropriate in‐vitro model is essential for accurate BCRP substrate characterization as well. The human BBB cell model, hCMEC/D3 cells, imitates the accurate BCRP expression levels found in primary human brain microvessels [33]. Literature reports consistently demonstrate increased intracellular levels of BCRP substrates in hCMEC/D3 cells following efflux inhibition studies, confirming the functionality of BCRP in these cells [34, 35, 36]. Hence, the apical localization of BCRP in hCMEC/D3 cells mimics its physiological role in efflux transport at the BBB [35]. Therefore, the present work aimed to investigate the impact of BCRP on the transport of phenytoin (PHT), carbamazepine (CBZ), valproic acid (VPA), N‐desmethyl clobazam (DCLB), lamotrigine (LTG), oxcarbazepine (OXC), topiramate (TPM), and levetiracetam (LEVI) using a multi‐in‐vitro approach utilizing ATPase, competitive substrate efflux, and bidirectional transport assays followed by sample quantification by high‐performance liquid chromatography (HPLC) or liquid chromatography/mass spectrometry (LC/MS) in hCMEC/D3 cells. Understanding the role of BCRP in limiting ASM levels in the brain is crucial for optimizing drug therapy, and may contribute to enhancing ASM efficacy in DRE cases.

## 2 Materials and Methods

### 2.1 Reagents and Chemicals

ASMs ‐ PHT (cat. no. PHR1139), CBZ (cat. no. C4024), VPA (cat. no. PHR1061), DCLB (cat. no. 18564), LTG (cat. no. L3791), OXC (cat. no. O3764), TPM (cat. no. T0575), LEVI (cat. no. L8668), mitoxantrone (MXR, cat. no. M6545), dimethyl sulfoxide (cat. no. 472301), and Ko143 (cat. no. K2144) were all purchased from Sigma‐Aldrich (St. Louis, MO, USA). Endothelial cell basal medium‐2 (cat. no. 190860) was bought from Lonza (Walkersville, MD USA); 1X Hanks’ balanced salt solution (cat. no. 14175079), sodium pyruvate (cat. no. 11360070), methanol (cat. no. 268280025), acetonitrile (cat. no. 26827), and chemically defined lipid concentrate (cat. no. 11905‐031), collagen type I (rat tail) (cat. no. A1048301) were purchased from Thermo Fisher Scientific (Waltham, MA, USA); 3‐(4,5‐dimethylthiazol‐2‐yl)‐2,5‐diphenyltetrazolium bromide (MTT; cat. no. 0793) was procured from Ameresco (Fountain Parkway, Solon, OH, USA); triton X‐100 (cat. no. T8787), hydrocortisone (cat. no. H0135), ascorbic acid (cat. no. PHR1008), and 4‐(2‐hydroxyethyl)‐1‐piperazine ethanesulfonic acid (HEPES; cat. no. PHR1428) were also obtained from Sigma‐Aldrich (St. Louis, MO, USA); formic acid (cat. no, 62673) was purchased from Sisco research laboratories Pvt. Ltd. (Mumbai, India); fetal bovine serum (cat. no. 10100‐147), penicillin‐streptomycin (cat. no. 15140122), basic fibroblast growth factor (cat. no. PHG0261), and BODIPY FL prazosin (BPZ, cat. no. B7433) were obtained from Invitrogen (Carlsbad, CA, USA).

### 2.2 Cell Culture

Human cerebral microvascular endothelial cells (hCMEC/D3; cat. no. CLU‐512) were bought from Cedarlane Laboratories (Burlington, Canada). Cells of passages below 40 were used for all the experiments except the ATPase assay. These were cultured with 5% CO_2_ at 37°C in endothelial cell basal medium‐2 containing 10 mM HEPES buffer, 1:100 chemically defined lipid concentrate, 1.4 μM hydrocortisone, 1 ng/ml basic fibroblast growth factor, 5 μg/ml ascorbic acid, 5% fetal bovine serum, and 1X antibiotic‐antimycotic solution, and seeded on collagen type I (rat tail)‐coated plates and flasks. MXR, PHT, CBZ, VPA, DCLB, LTG, OXC, TPM, Ko143, and BPZ were prepared in dimethyl sulfoxide and LEVI in nuclease free water. Notably, dimethyl sulfoxide not exceeding 0.5% was used as vehicle control in all the experiments.

### 2.3 MTT Cell Cytotoxicity Assay

The effect of ASMs, DCLB, and OXC on the viability of hCMEC/D3 cells was evaluated using MTT assay to determine non‐cytotoxic concentrations of ASMs. This assay was also used for determining the cytotoxicity of PHT, CBZ, VPA, LTG, TPM, and LEVI in hCMEC/D3 cells previously [37]. Briefly, cells were seeded at a density of 1 × 10^4^/well for 72 h on 96‐well plates coated with collagen type I and later, treated with a range of concentrations of respective ASMs. Assay was performed after 72 h treatment with the ASMs. To each well, 100 μL MTT solution (final concentration of 0.5 mg/ml) prepared in media was added and incubated for another 3 h at 37°C and 5%CO_2_. After 3 h, the formazan crystals were formed and dissolved with 100 μL dimethyl sulfoxide and measured 570 nm absorbance with 630 nm reference absorbance using Infinite 200 PRO NanoQuant (Tecan, Zurich, Switzerland). The results were obtained as absorbance of treated cells/absorbance of control cells x 100 and representative data is shown.

### 2.4 ATPase Assays

To assess the interaction between ASMs and BCRP, a vanadate‐sensitive ATPase assay was performed as per the manufacturer's instructions manual. Membrane preparations with overexpressed ABC transporters show the transport of compounds by ATP hydrolysis, hence this assay measures the ATPase activity by measuring the levels of inorganic phosphate produced by the colorimetric method that is modulated by the substrate drug of the transporter. The effect of DCLB and OXC on the BCRP ATPase activity was measured using SB BCRP M PREDEASY^TM^ ATPase assay kit (Solvo Biotechnology, Sigma‐Aldrich, Schnelldorf, Germany). Briefly, membrane vesicles from MCF‐7 cells overexpressing BCRP (procured from Solvo Biotechnology) were incubated for 10 min at 37°C with DMSO or water as the vehicle control or the various concentrations of DCLB or OXC with or without vanadate. To evaluate the effect of DCLB or OXC on the sulfasalazine‐stimulated BCRP ATPase activity, the membrane vesicles were treated with sulfasalazine, before incubation. After incubating with DCLB or OXC, each well was supplied with MgATP and incubated at 37°C for 10 min. After 10 min, 1x developer solution was added and kept for 2 min. To stop the reaction, 100 µL blocker solution was added and incubated for 30 min. at 37°C. The optical densities were measured at 620 nm using an Infinite 200 PRO NanoQuant (Tecan, Zurich, Switzerland) plate reader. 1 μM Ko143 was used as a positive control for BCRP inhibition. Ko143 was used at a concentration that is known to inhibit BCRP efficiently [35, 38]. The results were expressed as vanadate‐sensitive ATPase activity that is based on the difference in inorganic phosphate released between samples with and without vanadate. This will evaluate the ability of the test ASM to stimulate BCRP's baseline ATPase activity.

### 2.5 Competitive Substrate Efflux Assay

The potential of ASMs to compete with a known fluorescent substrate of BCRP, BPZ in hCMEC/D3 cells was examined using a competitive substrate efflux assay that has previously been used in various studies [34, 36, 37, 39, 40]. The interaction of CBZ, DCLB, OXC, TPM, and LEVI with BCRP was evaluated. Initially, hCMEC/D3 were seeded in 12‐well plates (cat. no. 150628, Nunc, Thermo Fisher Scientific, MA, USA) at 70‐80% confluency for 24 h. In this study, Ko143 was used for BCRP inhibition. The cultured cells were incubated at 37°C for half an hour in media containing BPZ with either the test ASMs or Ko143. Cells were washed with chilled 1X PBS three times and again incubated at 37°C for 1 h 30 min in BPZ‐free media with or without Ko143. Cells were washed three times with ice‐cold 1X PBS to terminate the assay. Cell lysis was performed by adding 1% triton X‐100, followed by incubating the cells for 15 min at 37°C. 20 µl of cell lysate was placed in 384‐well black assay plates for fluorescence detection (excitation at 488 nm; emission at 530 nm) to Infinite 200 PRO NanoQuant (Tecan, Zurich, Switzerland). The rest of the cell lysate was used for determining protein levels using the BCA protein estimation method to normalize the observed fluorescence intensity with total protein yield. The data were shown as a fold increment in intracellular fluorescence of BPZ relative to the control.

### 2.6 Bidirectional Transport Assay

For transport assays, hCMEC/D3 cells were seeded on collagen type I‐coated Transwell inserts (polycarbonate 12‐well, pore size 0.4 μM) (cat. no. CLS3460; Corning, NY, USA) at 80% confluency and cultured as described in section 2.5. In brief, the drug was dissolved in Hanks’ balanced salt solution supplemented with 10 mM HEPES, 1 mM Na‐Pyruvate, pH 7.4 at 37°C (transport buffer). Initially, both sides of the chamber were washed with 1X PBS and the assay was performed with pre‐warmed transport buffer. 0.5 ml or 1.5 ml of the drug solution, with or without BCRP‐specific inhibitor Ko143 (1 μM) was added to either the apical side or basolateral side of the monolayer, respectively, and fresh transport buffer was added to the opposite side. Aliquots of 100 μl samples were taken from the opposite sides every 30 min for 2 h, and the same amount of fresh transport buffer was added to the same chamber after each sampling. The bidirectional transport assays of positive control, MXR (5 μM), and all the ASMs under study at a therapeutic concentration range were performed as explained above. Concentrations of ASMs were evaluated by HPLC or LC/MS. Apparent permeability (P_app_) is defined as the transport rate of ASM and efflux ratio (ER) was calculated as the ratio of P_app_ in the basolateral‐to‐apical (P_app(B→A)_) direction to the P_app_ in the apical‐to‐basolateral (P_app(A→B)_) direction. The samples were stored at −20°C until analysis. The bidirectional transport assay of lucifer yellow (20 μM), a paracellular permeability marker of the human BBB cell model, hCMEC/D3 [41] was performed initially before each experiment to evaluate monolayer integrity and P_app_ as described by Wang et al., 2008 [42]. Fluorescence detection of lucifer yellow (excitation at 430 nm, and emission at 535 nm) was carried out with Infinite 200 PRO NanoQuant (Tecan, Zurich, Switzerland). Only the monolayers with integrity values that is P_app_ < 1 × 10^−3 ^cm/s as per published data were used [42, 43]. All transport assays were performed in triplicate.

### 2.7 Drug Analysis by HPLC/UV and LC/MS

PHT, CBZ, DCLB, LTG, and OXC were quantified by HPLC with UV detection as described earlier by Zhang et al., 2010 [44]. An HPLC/UV system (Waters, Milford, MA, U.S.A.) equipped with a 1525 solvent delivery module, a Reliant™ C18 HPLC column (5μm pores, 250 ×4.6 mm inner diameter), and a 2998 photodiode‐array UV detector was used. The mobile phase A consisted of 0.05% formic acid in water and acetonitrile and mobile phase B consisted of MilliQ water and 0.05% formic acid was used in all analyses reported here. The mobile phase flow rate was 1 mL/min and the ambient temperature was kept at 25°C. The resulting chromatograms were subsequently processed using Empower 2.0 software (Waters, India). Mobile phase A consisting of acetonitrile and 0.05% formic acid and mobile phase B consisting of MilliQ water and 0.05% formic acid were used in all analyses reported here. The optimized gradient pattern is as follows: 0–5 min, 20% B; 5–10 min, 50% B; 10–15 min, 80% B; and 15–20 min, 20% B. Retention time and detection wavelength were 10.4 min and 220 nm for PHT, 10 min and 285 nm for CBZ, 10.5 min and 229 nm for DCLB, 4.3 min and 270 nm for LTG, and 8.8 min and 258 nm for OXC. For both intraday and interday precision, the relative standard deviation (SD) for all the drugs was below 5%, as demonstrated in previously published literature [45, 46, 47, 48].

Quantification of VPA, and TPM was done by acquity UPLC H‐Class PLUS system coupled with xevo TQ‐S micro triple quadrupole mass spectrometer (Waters, India) and equipped with acquity BEH C18 column (100 mm, 1.7 uM, Waters India). The flow rate of mobile phase was set to 0.5 ml/min and the ambient temperature was kept at 25°C. The mobile phase A consisting of 0.02% ammonium hydrate in MQ water and mobile phase B consisting of 0.02% ammonium hydrate in acetonitrile was used in all analyses mentioned here. A gradient was applied using electrospray (ESI) in negative for VPA, and TPM, using the mobile phase described above. The gradient for the ESI‐ method started at 10% B, increased to 40% at min 0.5, to 80% at min 3.5, then to 98% at 3.75 min, and returned to initial conditions; 10% B at min 4.25, with a total time of 5 min. Retention times were 2.6, and 1.7 for VPA, and TPM respectively. The multiple reaction monitoring (MRM) transitions and MS conditions for the ASMs studied are provided in Table 1.

**Table 1:** MRM transitions, and MS parameters for each ASM detected by electrospray in negative (ESI–) mode.

ASM	S. no.	Parent Ion (*m/z*)	Fragment Ion (*m/z*)	Cone voltage (V)	Collision energy (eV)
VPA	1	143	143.1	15	5
	2	189	143	15	10
	3	287	143	15	10
TPM	1	335.03	77.5	30	40
	2	338.2	77.5	30	40
	3	338.2	78.2	30	40

### 2.8 Data Analysis

The calculation for apparent permeability coefficient, P_app_, was performed as described before. [42, 44]

$$P_{\text{app}}=\frac{\text{dQr}/\text{dt}}{A\times C_{0}}$$

where dQr/dt is the cumulative amount in the receiver side vs time in μM/s

A is the area of the cell monolayer

C_0_ is the initial concentration of the dosing solution (μM).

And,

$$\text{Efflux ratio}=\frac{P_{\text{app}}(B-\text{to}-A)}{P_{\text{app}}(A-\text{to}-B)}$$

All permeability calculations were performed in triplicate, and the data are shown as mean ± SD.

### 2.9 Statistical Analysis

All data were expressed as mean ± SD. Significant mean differences were calculated by using one‐way ANOVA with Tukey's HSD post‐hoc or Dunnett's post‐hoc test as indicated against each experimental result. Mean differences were considered significant statistically when *p* < 0.05(*) and *p* < 0.01(**).

## 3 Results

### 3.1 DCLB and OXC Stimulate ATPase Activity of BCRP

**Figure 1:**
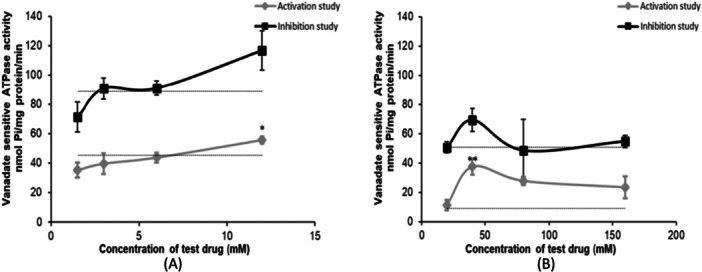
Effects of increasing concentrations of ASMs on vanadate‐sensitive BCRP ATPase activity. Vanadate‐sensitive activity in presence of (A) DCLB and (B) OXC in activation and inhibition experiments. The lower dotted line depicts the baseline vanadate‐sensitive ATPase, while the upper dotted line illustrates the activated ATPase induced by a reference substrate in the graphs. In the activation assays, increase in baseline ATPase activity (indicating the drug as a transporter substrate) were measured. Data are presented as mean ± SD obtained from three independent experiments. Statistically significant differences between respective controls and ASM‐treated activity were measured using one‐way ANOVA with Dunnett's post hoc test. (*p < 0.05, **p < 0.01).

To characterize whether test ASMs stimulate BCRP‐associated ATPase activity, we studied their effects over a concentration range on ATPase activity of BCRP‐overexpressing membrane vesicles. In the ATPase activation assay, DCLB and OXC significantly stimulated BCRP ATPase activity at 12 µM and 40 µM, respectively (Figure 1), suggesting potential interaction of DCLB and OXC with BCRP. In a previous study, we demonstrated that PHT, VPA, and LTG also stimulated BCRP ATPase activity, while no significant stimulation of BCRP ATPase activity was observed with CBZ, TPM, and LEVI [37].

### 3.2 DCLB and OXC Increase Intracellular Accumulation of BPZ

**Figure 2:**
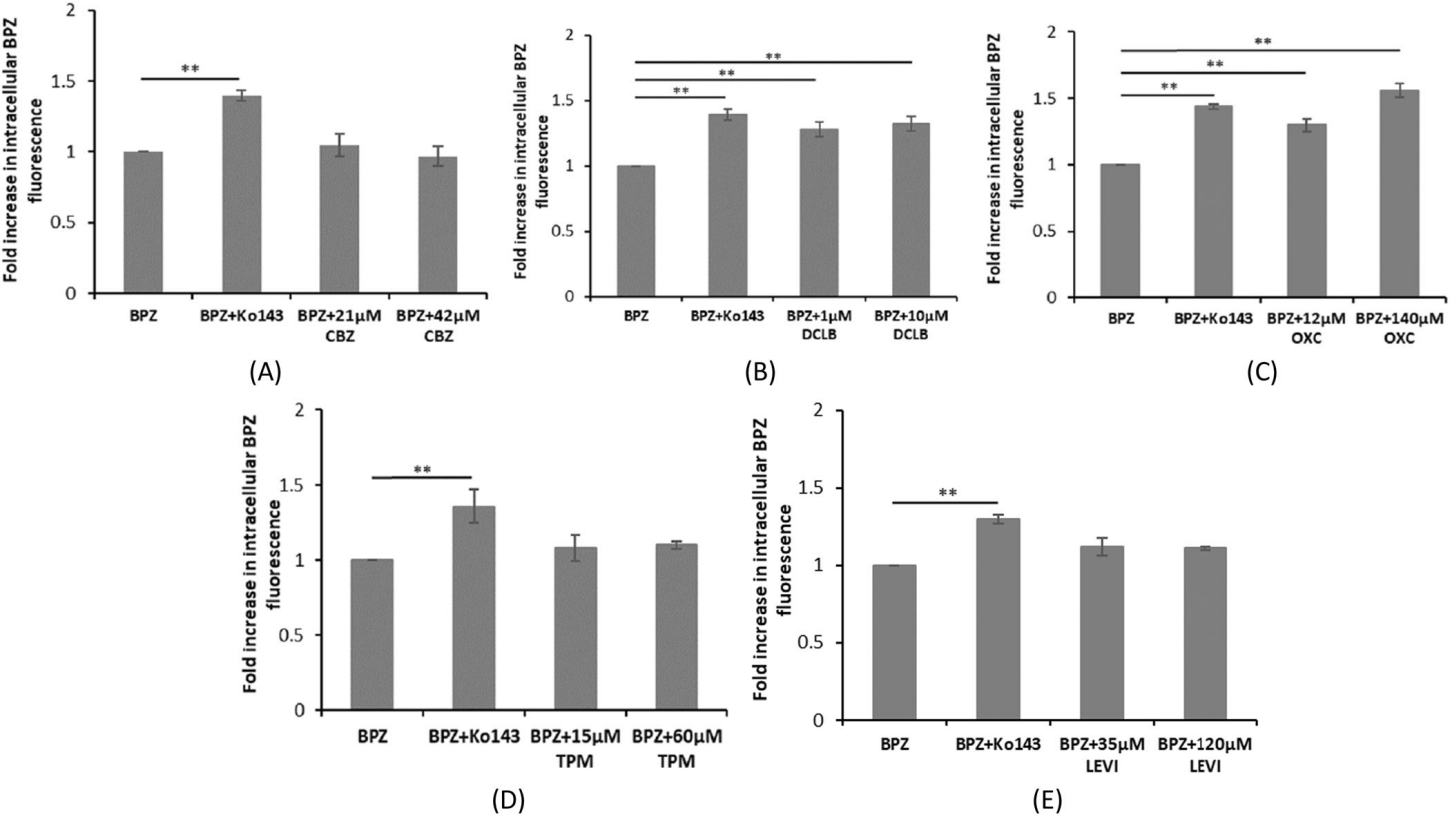
Effect of ASMs on the intracellular accumulation of Bodipy‐prazosin (BPZ) in hCMEC/D3 cells using competitive substrate efflux assay. Cells were treated with either ASM, (A) CBZ (B) DCLB (C) OXC (D) TPM (E) LEVI indicated, or Ko143, an inhibitor of BCRP. (BPZ, model substrate of BCRP) accumulation in cells was measured with or without Ko143 (1 μM). BPZ represents vehicle control (VC). The results are expressed as mean fluorescent intensity, normalized to the total protein concentration. Data is expressed as mean ± SD (*n* = 3) and statistical significance is determined using a one‐way ANOVA with post hoc Tukey's HSD test. (**p < 0.01).

Following confirmation of the absence of cytotoxicity of test ASMs on hCMEC/D3 cells at therapeutically relevant concentrations (Supplementary Table 1 and Supplementary Figure 1) [37], we studied the effect of test ASMs on the accumulation of BPZ (a BCRP fluorescent substrate) to investigate their interaction with BCRP. DCLB (1 and 10 µM respectively) and OXC (12 and 140 µM, respectively) significantly increased BPZ accumulation in hCMEC/D3 cells (Figure 2B and C, suggesting that DCLB and OXC may interact with BCRP. In our previous study, we showed via competitive substrate efflux assay that PHT, VPA, and LTG also interact with BCRP [37]. In this study, CBZ, TPM, and LEVI did not affect the intracellular accumulation of BPZ in hCMEC/D3 cells (Figures 2A,D, and E), indicating BCRP may not be involved in the transport of these ASMs.

### 3.3 Bidirectional Transport of VPA, DCLB, LTG, and OXC by BCRP

To confirm whether the test ASMs are effluxed out by BCRP, we evaluated their transport at two concentrations across polarized monolayers of hCMEC/D3 cells. In hCMEC/D3 cells, BCRP is located apically, hence, if the ASM ER ≥ 1.5 then it is considered directional transport and if it is significantly reduced upon BCRP inhibition then the transport is considered mediated by BCRP. Under the assay conditions, the ER of each ASM at the therapeutic concentration range with and without BCRP inhibitor Ko143 are summarized in Table 1.

**Figure 3:**
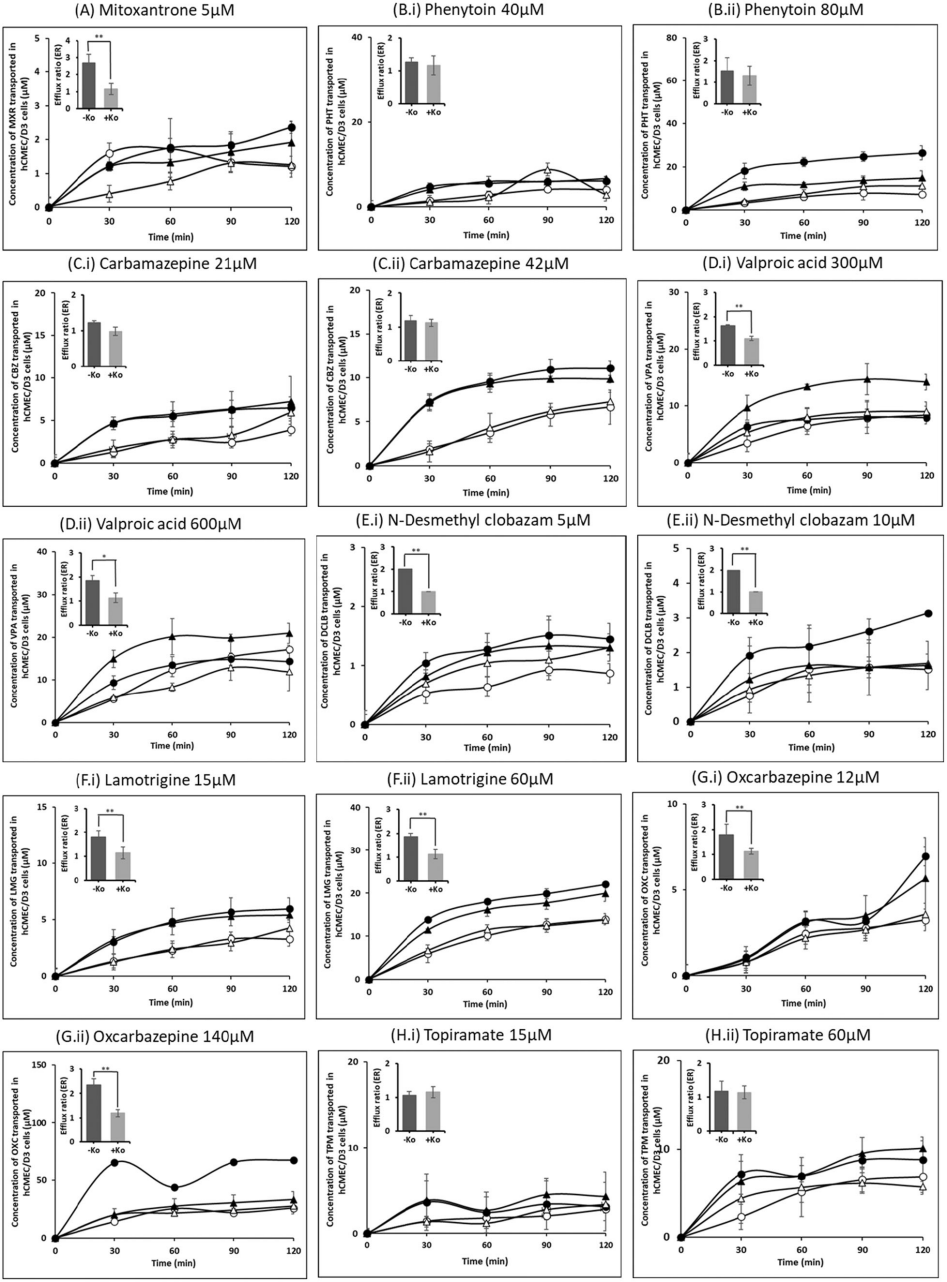
Bidirectional transport of all ASMs. (A). MXR, (B) (i‐ii) PHT; (C) (i‐ii) CBZ; (D) (i‐ii) VPA; (E) (i‐ii) DCLB; (F) (i‐ii) LTG; (G) (i‐ii) OXC; and (H) (i‐ii) TPM across monolayer of hCMEC/D3 cells with or without BCRP inhibitor Ko143. ● Basolateral‐to‐apical transport without BCRP inhibitor; ◯ apical‐to‐basolateral transport without BCRP inhibitor; ▲ basolateral‐to‐apical transport with BCRP inhibitor; △ apical‐to‐basolateral transport with BCRP inhibitor; 1 μM Ko143 was used as a specific inhibitor of BCRP. Ratios of ASM transport across cell monolayers (ASM transport in basolateral‐to‐apical direction divided by transport in apical‐to‐basolateral direction) with or without inhibitor were calculated using data acquired 2 h after ASM addition. Statistical significance between ER values of ASMs in the absence and presence of the inhibitor was calculated using unpaired student's t‐test (*p < 0.05, **p < 0.01), ‐Ko, absence of inhibitor; +Ko, presence of inhibitor. Data are represented as mean ± SD (*n* = 3).

MXR (known substrate of BCRP), served as a validating control in BCRP transport studies [49]. ER of MXR significantly decreased from 2.89 to 1.16 upon treatment with 1 μM Ko143 (a known inhibitor of BCRP) (Figure 3A, Table 1), demonstrating that MXR efflux is mediated by BCRP in hCMEC/D3 cells, hence validating the experimental model. VPA, DCLB, LTG, and OXC showed ERs above 1.5 at all the tested concentrations and displayed a decrease in the ER close to 1 in the presence of Ko143 (Figures 3D–G, Table 1). PHT showed an ER of 1.28 and 1.53 at 40 and 80 μM, respectively (Figure 3B), suggesting directional efflux at 80 μM. However, the decrease in ER to 1.31 at 80 μM due to the BCRP inhibitor was insignificant (Table 1). ER of CBZ and TPM at their respective concentrations was less than 1.5 and none of these ASMs showed any significant decrease in their ER in the presence of the Ko143 (Figures 3C and H). LEVI did not show any directional transport indicating that it may not be transported by BCRP (data not shown). These findings suggest that BCRP transports VPA, DCLB, LTG, and OXC, but not PHT, CBZ, TPM, and LEVI Table 2.

**Table 2:** P_app_ values, ER, and corrected efflux ratio (cER) of MXR and all ASMs in hCMEC/D3 cell monolayer. The P_app_ values of ASMs in the apical to basolateral and basolateral to apical directions were calculated using the equation described in section Materials and methods. The ER was calculated as P_app_ B‐A/P_app_ A‐B, and cER was calculated as the ER of ASMs without inhibitor divided by the ER of ASMs with inhibitor. Experiments were performed in triplicate (*n* = 3).

S. No.	ASM	Conc. (μM)	hCMEC/D3 without inhibitor Ko143 P_app_ x10^−6^ (cm/s)						hCMEC/D3 with inhibitor Ko143 P_app_ x10^−6^ (cm/s)						Corrected efflux ratio (cER)	
			A‐‐ > B		B‐‐ > A		Efflux ratio (ER)		A‐‐ > B		B‐‐ > A		Efflux ratio (ER)			
			Mean	SD	Mean	SD	Mean	SD	Mean	SD	Mean	SD	Mean	SD	Mean	SD
1	MXR	5	5.1	3.2	12.74	6.01	2.89	0.35	10.19	6.41	10.62	3	1.16	0.32	2.4	0.36
2	PHT	40	2.12	0.71	2.59	0.41	1.28	0.25	2.71	0.54	3.07	0.2	1.17	0.29	1.1	0.17
		80	3.09	2.12	3.36	1.25	1.53	0.6	2.03	0.37	2.12	0	1.31	0.45	1.17	0.15
3	DCLB	5	2.83	0	5.66	0	2	0	5.66	0	5.66	0	1	0	2	0
		10	2.83	0	5.66	0	2	0	2.83	0	2.83	0	1	0	2	0
4	LTG	15	4.67	0.87	8.71	2.02	1.87	0.23	3.69	2	3.93	1.66	1.14	0.25	1.67	0.31
		60	10.29	6.24	16.23	15.61	1.87	0.13	5.47	0.89	5.74	1	1.12	0.19	1.7	0.32
5	OXC	25	2.64	0.65	4.72	1.18	1.81	0.39	3.21	0.86	3.59	0.86	1.13	0.13	1.59	0.19
		140	16.51	1.43	39.4	7.48	2.37	0.26	139.66	15.59	168.91	38.64	1.2	0.15	1.98	0.03
6	VPA	300	0.19	0.07	0.31	0.1	1.62	0.04	0.21	0.1	0.24	0.13	1.1	0.09	1.49	0.16
		600	0.34	0.2	0.64	0.38	1.85	0.2	0.33	0.21	0.37	0.21	1.14	0.2	1.65	0.12
7	CBZ	21	3.37	1.17	4.27	2.37	1.23	0.41	4.72	2.7	3.03	1.43	0.98	0.12	1.23	0.32
		42	3.37	0.67	4.04	1.22	1.18	0.16	3.82	0.39	4.27	0.39	1.12	0.11	1.06	0.2
8	TPM	15	0.71	0	0.71	0	1.06	0.1	12.27	6.54	14.15	7.49	1.16	0.17	0.92	0.15
		60	19.99	6.26	25.48	7.51	1.17	0.29	158.53	8.01	189.67	24.02	1.13	0.19	1.03	0.2

## 4 Discussion

Resistance against ASM is a major challenge in epilepsy treatment and assessing their interaction with membrane‐bound drug efflux transporters may result in an effective therapeutic strategy [50, 51]. Since there is evidence of BCRP overexpression at brain epileptic foci in resected tissues from DRE patients [17, 20] and increased brain ASM concentrations in BCRP knockout mice [21, 30], therefore, studying BCRP‐mediated efflux of ASM from the brain is warranted. However, the present literature on BCRP‐mediated ASM efflux is either limited or inconclusive. Here, we used ATPase, competitive substrate efflux, and bidirectional transport assay to explore interactions of frequently prescribed ASMs, PHT, CBZ, VPA, DCLB, LTG, OXC, TPM, and LEVI with BCRP in‐vitro. ASMs were chosen based on their frequent clinical use and prior evidence of BCRP interactions [21, 24, 25, 52]. Since drug transport differs across compounds, each ASM's BCRP substrate status requires individual validation. The study showed that BCRP may cause efflux of VPA, DCLB, LTG, and OXC.

Our results showed that DCLB at 12 µM and OXC at 40 µM significantly increased BCRP‐specific ATP hydrolysis in ATPase assay (Figure 1) (*p* < 0.05 and *p* < 0.01 respectively), indicating that DCLB and OXC interact with BCRP. This was further supported by competitive substrate efflux assay where, DCLB (1 µM and 10 µM) and OXC (12 µM and 140 µM) significantly increased intracellular accumulation of BPZ in hCMEC/D3 cells (Figure 2) (*p* < 0.01). In bidirectional transport assay, DCLB (5 μM and 10 μM) and OXC (140 μM) exhibited an ER of 2 and showed a significant reduction in ER close to 1 when co‐incubated with BCRP inhibitor Ko143 (Figure 3) (*p* < 0.01), suggesting their BCRP‐mediated active efflux. This aligns with the International Transporter Consortium definition [32], which states that in epithelial cells, expressing efflux transporters, a compound with ER is ≥ 2, which is reduced by more than 50% in the presence of the transporter‐specific inhibitor is considered to be actively effluxed out by the transporter being studied. Previous in‐vivo study compared the brain distribution of ASMs between P‐gp knockout and P‐gp/BCRP dual gene knockout mice and revealed that BCRP contributes to restricting the brain distribution of clobazam [21], supporting our results of BCRP‐mediated DCLB (an active metabolite of clobazam) efflux. Interestingly, while OXC has been shown to be transported by P‐gp in a previous study [53], our results provided the first evidence of its BCRP‐mediated efflux.

Our previous data showed PHT, VPA, and LTG significantly stimulated BCRP ATPase activity in ATPase assay (*p* < 0.05) and increased BPZ accumulation in competitive substrate efflux assay conducted in hCMEC/D3 cells (*p* < 0.01), indicating their probable interaction with BCRP [37]. Therefore, in this study, we performed the bidirectional transport assay to further evaluate BCRP‐mediated transport of these ASMs. ER of PHT at 80 μM was 1.5, indicating weak directional efflux but there was no significant decrease in ER in the presence of Ko143 (Figure 3). Therefore, though PHT showed interaction with BCRP in ATPase and competitive substrate efflux assay [37], bidirectional transport assays did not show BCRP‐mediated efflux, hence the nature of interaction of PHT with BCRP remains in‐conclusive. Transport studies performed earlier in MDCKII cell line have also shown that PHT is not effluxed out by BCRP [24, 25], consistent with our findings. ER of VPA (300 μM and 600 μM), and LTG (15 μM and 60 μM) was greater than 1.5, showing weak directional transport, which was significantly reduced by Ko143 (Figure 3, *p* < 0.05), suggesting VPA and LTG are weakly transported by BCRP. A previous study using concentration equilibrium transport assay in MDCKII cell line showed that VPA is not transported by BCRP, which contradicts our findings [24, 25]. In case of LTG, one study reported it as a BCRP substrate using concentration equilibrium transport assay in MDCKII cell line [25], while another study in the same cell line stated it as non‐substrate [24]. The discrepancies of two independent studies observed in the same cell line underscore the limitations of using non‐brain‐derived cell lines, such as MDCKII, for accurately assessing the transport of ASMs by BCRP.

Our previous data indicated that CBZ, TPM, and LEVI did not stimulate BCRP ATPase activity in the ATPase assay [37]. Additionally, no increase in BPZ accumulation in the presence of CBZ, TPM, and LEVI, was observed in the competitive substrate efflux assay, suggesting that these ASMs may not interact with BCRP (Figure 2). The bidirectional transport assay showed that CBZ, TPM, and LEVI displayed ER less than 1.5 and no significant reduction was observed in the presence of Ko143, further supporting the lack of BCRP‐mediated transport of CBZ, TPM, and LEVI (Figure 3). Previous studies showed CBZ, TPM, and LEVI were not transported by BCRP using accumulation assays, bidirectional transport assays, and using concentration equilibrium transport assay in MDCKII, breast cancer cell line, and mouse embryonic fibroblast cell lines overexpressing BCRP [24, 25, 54], which supports our findings. However, an in‐vivo study revealed that BCRP limits the brain distribution of LEVI [21], which contradicts our findings.

Certain challenges were observed during this study, such as contradictions observed for VPA, LTG, and LEVI with previous studies regarding their interaction with BCRP [21, 24, 25] Therefore, further experimental validation is warranted utilizing more physiologically relevant models, like primary brain capillary endothelial cells, followed by validation in animal models using micro‐dialysis experiments. However, hCMEC/D3 cell line is a well‐established in‐vitro BBB model, valued for its high reproducibility, ease of culture, and human origin [43, 55], making it ideal for drug transport studies and allows for high‐throughput screening of substrate ASMs. Beyond its human origin, several key features make it a physiologically relevant system for studying BCRP‐mediated efflux, especially compared to widely used epithelial models like MDCKII cells. hCMEC/D3 cells express tight junction proteins, endothelial markers, and functional efflux transporters, including endogenous BCRP [36, 43, 56] which is critical for evaluating ASM permeability. In contrast, MDCKII cells lack BBB‐specific characteristics and require transfection to express BCRP, limiting their translational relevance [36, 57]. While human primary brain microvascular endothelial cells offer greater physiological relevance, they present significant limitations, including batch‐to‐batch variation, limited lifespan, and passage‐to‐passage phenotypic drift [58, 59, 60], which can affect experimental reproducibility. Thus, although primary cells could provide additional validation, the hCMEC/D3 model remains a reliable and practical initial choice for studying BBB permeability and drug transport mechanisms. In‐vitro model, hCMEC/D3 cells, plays a crucial role in early‐stage drug transport research, offering a controlled and reproducible system for evaluating BCRP‐mediated efflux. While they do not fully replicate the complexity of the BBB in‐vivo, they enable precise, cell‐specific analysis, which is essential before in‐vivo studies. hCMEC/D3 cells monolayer exhibits restricted permeability to lucifer yellow and various low molecular weight drugs, aligning with in‐vivo permeability coefficients [34, 35]. Additionally, studies confirm that hCMEC/D3 cells retain expression of key BBB transporters, including BCRP, supporting their relevance in drug transport research [43]. Given the challenges of isolating specific mechanisms in whole organisms, validated in‐vitro models serve as a foundational step in drug transport studies, providing valuable insights into epilepsy management. Despite some limitations, its permeability characteristics and transporter profile make it a robust in‐vitro model for investigating BBB mechanisms. However, further in‐vivo validation is necessary to confirm the therapeutic relevance of targeting BCRP. Moreover, certain ASMs such as VPA, LTG, and OXC are also transported by P‐gp, indicating they may have affinity for multiple transporters [28, 53, 61, 62]. Therefore, further investigation on the involvement of other transporters in ASM efflux and their interplay with BCRP is required.

## 5 Conclusion

We demonstrated that BCRP may mediate the efflux of VPA, LTG, DCLB, and OXC in hCMEC/D3 cells. DCLB and OXC stimulated BCRP ATPase activity and increased BPZ accumulation in hCMEC/D3 cells. Additionally, their efflux was significantly reduced by BCRP inhibitor in cell‐based bidirectional transport assay, confirming BCRP‐mediated transport of DCLB and OXC. VPA and LTG were previously shown to interact with BCRP and in this study via bidirectional transport assay, VPA and LTG are observed as weak BCRP substrates. While initial findings from the ATPase assay and competitive substrate efflux assay suggested a potential interaction between PHT and BCRP, however, it remains unclear whether PHT is actually transported by BCRP, since the results of bidirectional transport assay did not demonstrate a decrease in its ER on BCRP inhibition. The remaining ASMs, CBZ, TPM, and LEVI, did not show any significant interaction with BCRP, however, further investigation is needed to definitively confirm their nature of interaction with BCRP. Therefore, these findings suggest that targeting BCRP may be a potential therapeutic strategy to enhance the efficacy of VPA, LTG, DCLB, and OXC in DRE patients.

## Author Contributions

**Shivangi Bora:** data curation, formal analysis, investigation, methodology, validation, writing – original draft, writing – review and editing. **Priyanka Rani Paul:** data curation, formal analysis, investigation, writing – review and editing. **Samiksha Kukal:** methodology, visualization, writing – review and editing. **Manish Kumar Mishra:** writing – review and editing. **Yasha Hasija:** visualization, supervision, writing – review and editing. **Ritushree Kukreti:** conceptualization, funding acquisition, project administration, resources, supervision, validation, visualization, writing – review and editing.

## Ethics Statement

The authors have nothing to report.

## Consent

The authors have nothing to report.

## Consent to Publish

This article does not contain any result/data/figure that is published or under consideration for publication.

## Conflicts of Interest

The authors declare no conflicts of interest.

## Supporting information


**Supplementary Figure 1:** Cytotoxicity of ASMs in hCMEC/D3 Cell line using MTT assay. MTT assay was performed with (i) N‐Desmethyl clobazam and (ii) Oxcarbazepine in hCMEC/D3 cells. Cells (10,000 cells/well) were plated in 96‐well plates for 24hr and subsequently treated with varying concentrations of drugs for 72hr.

**Supplementary Table 1:** Therapeutic plasma concentrations (reference range) of Antiseizure medications in patients with epilepsy (Johannessen, 2004; Patsalos, 2008; Lee, 2016).

## Data Availability

Data will be made available on request from the authors.
