# Supplementary material for: In‐vitro Assessment of BCRP‐Mediated Efflux of Antiseizure Medications in Human Blood‐Brain Barrier Cell Model
Source: J Biochem Mol Toxicol. 2025 Oct 27;39(11):e70570. doi: 10.1002/jbt.70570 (PMC12555108; doi:10.1002/jbt.70570)
Supplement: Supplementary file 1 — Supplementary Figure 1: Cytotoxicity of ASMs in hCMEC/D3 Cell line using MTT assay. MTT assay was performed with (i) N‐Desmethyl clobazam and (ii) Oxcarbazepine in hCMEC/D3 cells. Cells (10,000 cells/well) were plated in 96‐well plates for 24hr and subsequently treated with varying concentrations of drugs for 72hr. [file JBT-39-e70570-s001.docx]

**
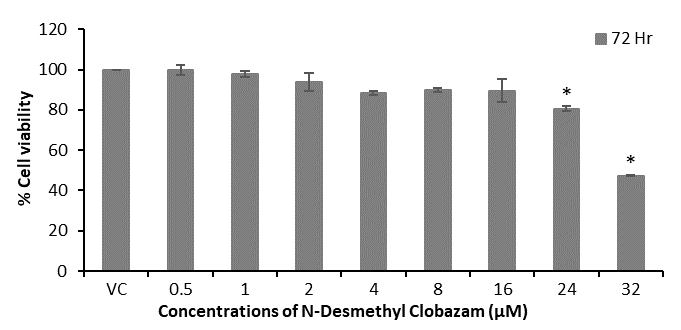
**

**(i)**

**
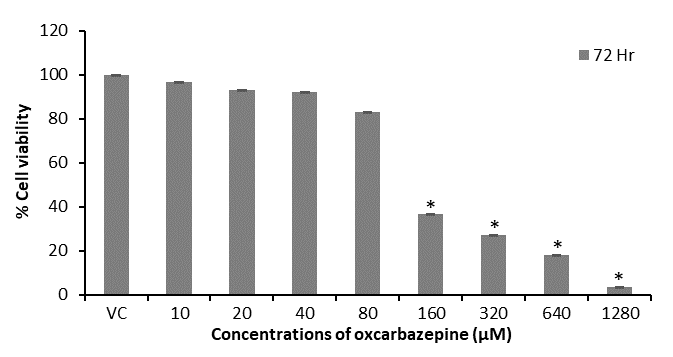
**

**(ii)**

**Supplementary fig. 1:** **Cytotoxicity of ASMs in hCMEC/D3 Cell line using MTT assay.** MTT assay was performed with (i) N-Desmethyl clobazam and (ii) Oxcarbazepine in hCMEC/D3 cells. Cells (10,000 cells/well) were plated in 96-well plates for 24hr and subsequently treated with varying concentrations of drugs for 72hr. Data represents the mean ± SD of five (n=5) independent experiments. Statistical significance (*, p<0.05) was determined using one-way ANOVA with Dunnet’s post-hoc test. Non-cytotoxic and therapeutic doses were used for further experiments in the study. Doses encircled in red highlight the therapeutic concentration range
