# Supplementary material for: In‐vitro Assessment of BCRP‐Mediated Efflux of Antiseizure Medications in Human Blood‐Brain Barrier Cell Model
Source: J Biochem Mol Toxicol. 2025 Oct 27;39(11):e70570. doi: 10.1002/jbt.70570 (PMC12555108; doi:10.1002/jbt.70570)
Supplement: Supplementary file 2 — Supplementary Table 1: Therapeutic plasma concentrations (reference range) of Antiseizure medications in patients with epilepsy (Johannessen, 2004; Patsalos, 2008; Lee, 2016). [file JBT-39-e70570-s002.docx]

**Supplementary table 1:** Therapeutic plasma concentrations (reference range) of Antiseizure medications in patients with epilepsy (Johannessen, 2004; Patsalos, 2008; Lee, 2016)

| **Antiseizure medication** | **Therapeutic plasma concentration range (μM)** | **Concentrations used**  **in the present study** |
| --- | --- | --- |
| Phenytoin | 40-80 | 40μM, 80μM |
| Carbamazepine | 15-45 | 21μM, 42μM |
| Valproic acid | 300-600 | 300μM, 600μM |
| N-desmethyl clobazam | 1-10 | 1μM, 5μM, 10μM |
| Lamotrigine | 10-60 | 15μM, 60μM |
| Oxcarbazepine | 12-140 | 12μM, 25μM, 140μM |
| Topiramate | 15-60 | 15μM, 60μM |
| Levetiracetam | 35-120 | 40μM, 120μM |

**References**

1. Johannessen SI, Battino D, Berry DJ, Bialer M, Krämer G, Tomson T, Patsalos PN. Therapeutic drug monitoring of the newer antiepileptic drugs. Ther Drug Monit. 2003 Jun;25(3):347-63. doi: 10.1097/00007691-200306000-00016.
2. Lee CY, Lai HY, Chiu A, Chan SH, Hsiao LP, Lee ST. The effects of antiepileptic drugs on the growth of glioblastoma cell lines. J Neurooncol. 2016 May;127(3):445-53. doi: 10.1007/s11060-016-2056-6. Epub 2016 Jan 13.
3. Patsalos PN, Berry DJ, Bourgeois BF, Cloyd JC, Glauser TA, Johannessen SI, Leppik IE, Tomson T, Perucca E. Antiepileptic drugs--best practice guidelines for therapeutic drug monitoring: a position paper by the subcommission on therapeutic drug monitoring, ILAE Commission on Therapeutic Strategies. Epilepsia. 2008 Jul;49(7):1239-76. doi: 10.1111/j.1528-1167.2008.01561.x.
